# Teaching science content in nursing programs in Australia: a cross-sectional survey of academics

**DOI:** 10.1186/s12912-015-0074-x

**Published:** 2015-05-01

**Authors:** Melanie Birks, Nicholas Ralph, Robyn Cant, Elspeth Hillman, Ylona Chun Tie

**Affiliations:** Centre for Nursing and Midwifery Research, College of Healthcare Sciences, James Cook University, Townsville, QLD Australia; Centre for Health Sciences Research, School of Nursing and Midwifery, University of Southern Queensland, Toowoomba, QLD Australia; School of Nursing and Midwifery, Monash University, Melbourne, VIC Australia; College of Healthcare Sciences, James Cook University, Townsville, QLD Australia

**Keywords:** Bioscience, Curriculum content, Curriculum design, Nursing, Science, Undergraduate nursing education

## Abstract

**Background:**

Professional nursing practice is informed by biological, social and behavioural sciences. In undergraduate pre-registration nursing programs, biological sciences typically include anatomy, physiology, microbiology, chemistry, physics and pharmacology. The current gap in the literature results in a lack of information about the content and depth of biological sciences being taught in nursing curricula. The aim of this study was to establish what priority is given to the teaching of science topics in these programs in order to inform an understanding of the relative importance placed on this subject area in contemporary nursing education.

**Method:**

This study employed a cross-sectional survey method. This paper reports on the first phase of a larger project examining science content in nursing programs. An existing questionnaire was modified and delivered online for completion by academics who teach science to nurses in these programs. This paper reports on the relative priority given by respondents to the teaching of 177 topics contained in the questionnaire.

**Results:**

Of the relatively small population of academics who teach science to nursing students, thirty (n = 30) completed the survey. Findings indicate strong support for the teaching of science in these programs, with particular priority given to the basic concepts of bioscience and gross system anatomy. Of concern, most science subject areas outside of these domains were ranked as being of moderate or low priority.

**Conclusion:**

While the small sample size limited the conclusions able to be drawn from this study, the findings supported previous studies that indicated inadequacies in the teaching of science content in nursing curricula. Nevertheless, these findings have raised questions about the current philosophy that underpins nursing education in Australia and whether existing practices are clearly focused on preparing students for the demands of contemporary nursing practice. Academics responsible for the design and implementation of nursing curricula are encouraged to review the content of current programs in light of the findings of this research.

## Background

Developing a strong foundation of biological science (or bioscience) knowledge in nursing students is an important aspect of their preparation for practice. Clinical environments are increasingly characterised by escalating patient acuity; advancing technology and treatment modalities; and complex requirements in managing current and emerging health priorities [1-3]. Adequate preparation for the professional role is therefore dependent on strong theoretical foundation. Contemporary nursing practice is informed by bioscience, along with social and behavioural sciences [4,5]. Bioscience in undergraduate pre-registration nursing programs typically includes anatomy, physiology, microbiology, chemistry, physics and pharmacology [6].

Over the last thirty years, an increasing body of research has highlighted the need for registered nurses (RNs) to have a sound knowledge of bioscience in order to recognise, comprehend, and respond safely and competently to changes in a patient’s physiological and pathophysiological health status [1,2,4,7-12]. In particular, having a strong emphasis on human anatomy and physiology in preparatory nursing education ensures a foundation in the sciences that facilitates safe and effective nursing assessment at the point-of-care [2,4,7,8].

While there is consensus within the literature regarding the need for and importance of bioscience in nursing curricula, the accreditation requirements for nursing programs in Australia do not specifically prescribe bioscience as an essential aspect of the educational experience [13]. Nursing curricula is already crowded with what is deemed essential content [14]. Thus nurse academics are challenged to ensure that relevant bioscience is integrated into curricula to produce graduates who synthesise and integrate this knowledge in the applicable clinical contexts [2,4,15]. Although there are studies on how bioscience should be taught, who is best placed to teach bioscience, and the challenges students face in learning bioscience [7,16-21], there is no evidence of any cohesive or widespread approach to facilitate the teaching of bioscience in nursing in Australia.

Further difficulties in successfully teaching bioscience content in nursing programs were experienced as students reportedly viewed bioscience subjects with trepidation as they perceived it to be the most difficult aspect of the nursing program [7]. Similarly, a lack of confidence among RNs in applying bioscience knowledge clinically compounded this issue [1,2,22] as they felt they did not possess the ability to synthesis and integrate bioscience knowledge into their clinical practice [7,18,19,23].

That a lack of confidence exists in students *and* RNs in establishing knowledge linkages between bioscience in theory and practice raises questions about the quality of learning and teaching strategies employed. However, several strategies for teaching bioscience content were evident in the literature. A clear emphasis on stimulating the learning of bioscience content through the use of clinically relevant case studies and examples was encouraged [7,16]. Using a variety of online resources has showed promise with two studies reporting improved student performance through the implementation of supportive computer-based tools designed to stimulate practical learning [7,21]. Although improved resourcing was seen to be important, the need for nurse academics and scientists to collaborate was identified in order to integrate bioscience content more meaningfully in curricula.

Despite bioscience being a fundamental component of Australian nursing education programs since the early 1990s [24], only a handful of studies have offered empirically-driven strategies that can improve approaches to learning and teaching. As a result, there is an ongoing lack of research on the relevance of the content, depth and quality of bioscience teaching and learning in nursing curricula. The research undertaken in this paper aims to establish what science topics are taught in undergraduate nursing programs in Australia and what priority is given to the teaching of this content.

## Method

### Study design

A cross-sectional survey design was employed in this research to address the aim of this research. This study was part of a broader nation-wide project that was delivered in two phases. The results presented in this paper are drawn from the first phase, in which a population of academics who taught science in nursing programs were asked to identify the priority given to various topics taught in these programs. The second phase of the study involved a survey of registered nurses to ascertain what priority they believe should be given to the same content. Results from that subsequent phase will be reported elsewhere.

### Instrument

This study employed an online survey, modified from a questionnaire developed by Logan [25]. The original questionnaire content was determined from program documentation, accreditation guidelines and literature, with the actual survey design being informed by focus group discussion [18]. The questionnaire was adapted for the current study to ensure clarity of terminology, flow and relevance to the project aims. The original questionnaire had been used to survey 81 registered nurses in a mixed methods study, however no statistical reliability data were given in the original work. The final instrument used in the current study comprised 177 science topics clustered into the following categories: Normal gross anatomy of body systems (11 items); Basic concepts (6); Normal cellular histology (10); Physiology and pathophysiology of body systems (86); Microbiology (22); Chemistry (20); and Physics (24). Pharmacology was excluded from this survey as it was deemed to be an area of specialized subject matter.

Each item was rated on a 5-point scale of priority from 1 (low priority) to 5 (highest priority). These science topics were accompanied by 10 questions about personal demographics (including respondents’ age and residential postcode) and opportunity for open text comments. Members of the research team and six other nursing academics from various institutions tested the survey to ensure internal validity. A number of refinements were made to language and functionality in response to feedback following testing and prior to the survey being posted online via a subscription survey site.

### Sample

This study sought to establish what priority is giving to the teaching of various science topics in nursing programs in Australia. Input was therefore sought from academics who taught this content. With these teaching positions being subject to approval by the Australian Nursing and Midwifery Accreditation Council [13], minimum qualifications and experience was assured. Academics teaching in graduate entry degrees were excluded from this study given the variable entry requirements for those programs. An invitation to participate in the survey was sent via the Council of Deans of Nursing and Midwifery (Australia and New Zealand) (CDNM). The CDNM represents Deans and heads of schools of nursing from “universities that offer undergraduate and postgraduate nursing programs across Australia and New Zealand” [26]. The CDNM sent the invitation to the 35 Australian based member institutions, asking Deans and heads of school to disseminate the invitation to staff who met the inclusion criteria. Each institution varies in respect of the number of academics who teach science content to nurses, and whether this role is undertaken by nursing or science faculty. The total target population is therefore in flux, however the research team estimated that at least two persons who meet the inclusion criteria would be employed at each institution.

### Data collection and analysis

Following approval from the James Cook University Human Research Ethics Committee, a link to the online survey was disseminated to academic staff via members of the CDNM. Consent was implied by return of the completed survey questionnaire. Demographic data and responses to science content topic areas collected using this tool were subjected to analyses. IBM-SPSS 22 (IBM Corp., Armonk, New York, 2011) was used to conduct analysis using descriptive and inferential statistics. The Mann-Whitney *U* test was used to test for differences between groups, with p < 0.05 regarded as significant. These results are presented in the following section.

## Results

### Demographics

Thirty academics who taught science to nurses participated in this survey. With the exception of Western Australia, respondents were distributed across all Australian states and territories. Broad representation was therefore secured from across the sector. Around half reported they were registered nurses (16/30). Most respondents held a postgraduate degree with nine nurses and nine others from non-nursing disciplines holding a doctoral degree. The majority (*n* = 25) were aged over 40 years of age, with an average science teaching experience of 8.4 years (range: 1-25). Nurse academics identified their specialties as mainly in nursing, including in emergency and critical care; medical-surgical nursing; pathophysiology and pharmacology; education; and medical science. Specialties identified by non-nursing respondents were various and included biological sciences such as physiology, pathology, neurology, pharmacology, immunology, anatomy and medical science.

### Priority of science topics

Nursing academics and non-nursing academics who responded to the survey were strongly supportive of the teaching of all science topics. Overall, when rated on a scale of 1 (least priority) to 5 (highest priority) no individual item received an average rating of less than 2.4. Whilst this equates to confirmation that all the surveyed science topics should be taught, there was variation between individual items. Tables 1, 2, 3, 4, 5 and 6 present each item and mean ratings. For clarity of interpretation, items have been categorized in accordance with broad subject areas and divided into topics rated as ‘High priority’ (those that have a mean ranking of 4 and above), ‘Moderate priority’ (those that have a mean ranking of 3-3.99) and ‘Low priority’ (those that have a mean ranking below 3).

**Table 1:** **Basic concepts, anatomy and histology topics ranked as high priority (4 or above) by academics** ***(ranking: 1 = lowest priority to 5 = highest priority)***

**Topic**	**Mean/SD**	**Range**
***Basic concepts***		
Homeostasis	4.72 ± 0.53	3-5
Tissue types and functions	4.15 ± 0.83	2-5
Surface anatomy, body planes, orientation terminology	4.10 ± 0.94	2-5
***Normal gross anatomy of:***		
Cardiovascular system	4.72 ± 0.53	3-5
Respiratory system	4.72 ± 0.53	3-5
Neurological system	4.66 ± 0.55	3-5
Renal system	4.55 ± 0.63	3-5
Gastrointestinal system	4.41 ± 0.73	3-5
Immune system	4.21 ± 0.98	1-5
Endocrine system	4.24 ± 0.95	1-5
Muscular system	4.00 ± 1.08	1-5
Skeletal system	4.00 ± 0.85	2-5
Integumentary system	4.00 ± 0.78	2-5
***Normal cellular histology of:***		
Respiratory system: trachea, bronchi, bronchioles, alveoli	4.21 ± 1.07	1-5
Neurological system: neurons and neuroglia	4.11 ± 0.92	1-5

**Table 2:** **Physiology and pathophysiology topics ranked as high priority (≥4) by academics** ***(ranking: 1 = lowest priority to 5 = highest priority)***

**Topic**	**Mean/SD**	**Range**
***Normal physiology and pathophysiology of:***		
**Cardiovascular system**		
Blood composition and haemostasis	4.00 ± 0.00	2-4
Myocardial ischaemia and infarction	4.00 ± 0.00	4-4
**Respiratory system**		
Gas exchange	4.00 ± 0.00	4-4
**Renal system**		
Body fluids and water balance	4.00 ± 0.00	4-4
Electrolytes	4.00 ± 0.00	4-4
Urine production and excretion	4.00 ± 0.00	4-4
**Genetics**		
Cell differentiation, proliferation with particular reference to genetic control and malignancy	4.47 ± 0.70	2-4

**Table 3:** **Basic concepts, anatomy, and histology topics ranked as moderate priority (3 – 3.99) by academics** ***(ranking: 1 = lowest priority to 5 = highest priority)***

**Topic**	**Mean/SD**	**Range**
***Basic concepts***		
Generic cell structure	3.90 ± 0.98	2-5
Biology of cancer	3.31 ± 1.26	1-5
Organelle function	3.21 ± 1.01	1-5
***Normal gross anatomy of:***		
Reproductive system	3.83 ± 0.97	2-5
***Normal cellular histology of:***		
Immune system: lymph nodes, spleen, marrow, blood cells	3.89 ± 1.26	1-5
Gastrointestinal: liver, pancreas, salivary, biliary, alimentary canal	3.89 ± 0.99	1-5
Muscle types: cardiac, smooth and skeletal	3.75 ± 1.08	1-5
Renal system: nephrons, ureter, bladder, urethra	3.54 ± 0.52	3-4
Skeletal: bone, cartilage	3.50 ± 1.44	1-5
Endocrine system: glandular tissue	3.47 ± 0.69	2-4
Integument – thick and thin skin	3.43 ± 0.88	1-5
Reproductive system: ovary, testes, uterus, vagina	3.29 ± 1.12	1-5

**Table 4:** **Physiology and pathophysiology topics ranked as moderate priority (3 – 3.99) by academics** ***(ranking: 1 = lowest priority to 5 = highest priority)***

**Topic**	**Mean/SD**	**Range**
***Normal physiology and pathophysiology of:***		
**Musculoskeletal system**		
Joint structure and function	3.53 ± 0.61	2-4
Muscle contraction	3.52 ± 0.60	2-4
Bone tissue – formation and growth	3.46 ± 0.66	2-4
Fracture and fracture repair	3.45 ± 0.61	2-4
Major infectious musculoskeletal diseases	3.33 ± 0.66	2-4
Locomotion	3.30 ± 0.70	2-4
**Integument**		
Major infections and diseases of integumentary system	3.63 ± 0.60	2-4
Wounds, burns and healing	3.50 ± 0.80	2-4
Formation and growth	3.21 ± 0.59	2-4
**Cardiovascular system**		
Vessels: heart, arteries, veins and lymph	3.88 ± 0.35	3-4
Peripheral resistance	3.83 ± 0.39	3-4
Cardiac output, Stroke volume	3.82 ± 0.40	3-4
Blood pressure and BP measurement	3.67 ± 0.81	2-4
Lymph nodes and lymph organs	3.55 ± 0.67	2-4
Major infections and diseases of cardiac system integumentary system	3.53 ± 0.74	2-4
Baroreceptor reflex	3.50 ± 0.52	3-4
Cardiac markers and disease	3.45 ± 0.82	2-4
Frank Starling Law of the Heart	3.41 ± 0.51	2-4
Foetal circulation changes	3.18 ± 0.66	2-4
Respiratory system		
Respiratory control	3.91 ± 0.30	3-4
Breathing mechanics	3.83 ± 0.39	3-4
Blood gas transport	3.78 ± 0.44	3-4
Haemoglobin – oxyhaemoglobin curve	3.73 ± 0.46	3-4
Major infections and diseases of respiratory system	3.69 ± 0.63	2-4
COPD and oxygen therapy	3.64 ± 0.51	3-4
**Respiratory volumes - spirometry**	3.60 ± 0.60	2-4
**Gastro-intestinal system**		
Liver and gallbladder	3.87 ± 0.35	3-4
Pancreas	3.85 ± 0.38	3-4
Role of hormones	3.65 ± 0.70	2-4
Digestion, absorption and metabolism	3.58 ± 0.52	3-4
Major infections and gastrointestinal diseases	3.57 ± 0.65	2-4
Major nutrients – food pyramid: proteins, carbohydrates and lipids	3.53 ± 0.63	2-4
Glucose metabolism and ATP production	3.47 ± 0.74	2-4
Enzymes	3.47 ± 0.62	2-4
Phases of digestion	3.39 ± 0.70	2-4
Salivary glands	3.22 ± 0.74	2-4
**Neurological system**		
ANS – parasympathetic and sympathetic	3.90 ± 0.32	3-4
PNS – reflexes, sensory receptors	3.77 ± 0.44	3-4
CNS – brain and spinal cord	3.73 ± 0.47	3-4
Synapses and neuromuscular function	3.72 ± 0.58	2-4
Major infections and neurological diseases system	3.72 ± 0.58	2-4
CSF production and flow	3.59 ± 0.59	2-4
Pain	3.57 ± 0.85	2-4
Membrane potentials, action potentials neurotransmitters	3.56 ± 0.63	2-4
Fight and flight response	3.55 ± 0.52	3-4
Integrative properties of neurons	3.40 ± 0.60	2-4
Neuroglia	3.38 ± 0.75	2-4
Special senses: taste, vision, hearing, balance, smell	3.38 ± 0.74	2-4
**Reproductive system**		
Male and female	3.74 ± 0.56	2-4
Endocrine control	3.72 ± 0.46	3-4
Major infections/diseases of the reproductive system	3.50 ± 0.62	2-4
Pregnancy	3.42 ± 0.77	2-4
Contraception	3.38 ± 0.74	2-4
Embryonic and foetal development	3.30 ± 0.70	2-4
Parturition and foetal changes at birth	3.19 ± 0.68	2-4
Fertilization, implantation	3.13 ± 0.82	2-4
Fertility	3.05 ± 0.67	2-4
**Renal system**		
Normal and abnormal constituents of urine	3.91 ± 0.30	3-4
Acidosis and alkalosis	3.82 ± 0.40	3-4
Renal dialysis	3.61 ± 0.58	2-4
Major infections and diseases of renal system the renal system	3.59 ± 0.71	2-4
**Endocrine system**		
Homeostasis	3.86 ± 0.38	3-4
Hormone control, neuroendocrine axis	3.83 ± 0.39	3-4
Biology of cancer	3.59 ± 0.62	2-4
Mechanisms of hormone release	3.53 ± 0.51	3-4
**Immune system**		
Non-specific and specific body defenses	3.92 ± 0.29	3-4
Active and passive immunity	3.83 ± 0.39	3-4
Infection and biological response	3.70 ± 0.68	2-4
Autoimmune disease	3.60 ± 0.60	2-4
Humoral and cell-mediated immunity	3.56 ± 0.63	2-4
Immunodeficiency	3.55 ± 0.51	3-4
Hypersensitivity	3.50 ± 0.51	3-4
Antigens	3.31 ± 0.60	2-4
Tissue transplantation	3.00 ± 0.74	2-4
**Genetics**		
Human genetic disorders, principles of screening	3.50 ± 0.61	2-4
Coding from DNA to protein production	3.33 ± 0.77	2-4
Basic patterns of human inheritance	3.40 ± 0.60	2-4
DNA genes and chromosome structure	3.29 ± 0.64	2-4
Mitosis and meiosis – development of ova and sperm	3.05 ± 0.67	2-4

**Table 5:** **Microbiology, chemistry and physics topics ranked as moderate priority (3 – 3.99) by academics** ***(ranking: 1 = lowest priority to 5 = highest priority)***

**Topic**	**Mean/SD**	**Range**
**Microbiology**		
Bacteria	3.87 ± 0.35	3-4
Viruses	3.80 ± 0.41	3-4
Control of microbial growth	3.75 ± 0.56	2-4
Disease transmission	3.73 ± 0.65	2-4
The role of antiseptic, soap and alcohol	3.73 ± 0.48	3-4
Common infectious diseases	3.67 ± 0.65	2-4
Vaccines	3.57 ± 0.76	2-4
Fungi	3.55 ± 0.69	2-4
Control of microbial growth	3.53 ± 0.74	2-4
Chemical and physical requirements for microbial growth	3.53 ± 0.51	2-4
Virulence and resistance	3.50 ± 0.76	2-4
Asepsis	3.50 ± 0.76	2-4
Principles of epidemiology	3.48 ± 0.75	2-4
Reservoirs and vectors (e.g. mosquitos)	3.40 ± 0.82	2-4
Protozoans	3.40 ± 0.60	2-4
Helminths (worms)	3.33 ± 0.84	2-4
History and scope of microbiology	3.33 ± 0.77	2-4
Microbiological tools, techniques and procedures	3.29 ± 0.85	2-4
Eukaryotics and prokaryotics	3.29 ± 0.77	3-4
Relationship of microbes and cancer	3.27 ± 0.76	2-4
Biotechnology	3.15 ± 0.81	2-4
Algae	3.11 ± 0.83	2-4
**Chemistry**		
Electrolytes	3.88 ± 0.34	3-4
pH as a scale of acidity, buffers, body buffer systems and blood gases	3.73 ± 0.46	3-4
Chemical symbols – e.g. Na, K, Ca	3.73 ± 0.59	2-4
Diffusion	3.69 ± 0.48	3-4
Osmosis	3.69 ± 0.48	3-4
Concentration, solubility in making solutions, solutions and body fluids	3.65 ± 0.61	2-4
Protein, carbohydrate and lipid chemical structures	3.65 ± 0.61	2-4
Hydration	3.62 ± 0.51	3-4
Filtration	3.56 ± 0.62	2-4
Catalysts, enzymes	3.47 ± 0.70	2-4
Anaerobic and aerobic respiration	3.46 ± 0.66	2-4
Catabolism and Anabolism	3.41 ± 0.67	2-4
Production of ATP	3.35 ± 0.75	2-4
Citric acid cycle (Krebs cycle) and electron transport chain	3.29 ± 0.78	2-4
Periodic table	3.20 ± 0.78	2-4
Covalent, ionic, and hydrogen bonds	3.11 ± 0.81	2-4
Organic chemistry – alkanes, alkenes, alkyls, alcohols	3.05 ± 0.85	2-4
Chemical equations	3.05 ± 0.78	2-4
Chemical change	3.00 ± 0.84	2-4
**Physics**		
Measurement – quantities	3.56 ± 0.88	2-4
Temperature and heat transfer	3.56 ± 0.71	2-4
Fluid flow: length/radius of tube; viscosity; resistance; flow rate; venturi effect	3.50 ± 0.69	2-4
Measurement - relationship of volume to weight	3.44 ± 0.63	2-4
Measurement - errors	3.33 ± 0.89	2-4
Pressure, pressure in fluids, pressure gradients and pressure between solids	3.30 ± 0.87	2-4
Measurement - graphing	3.27 ± 0.88	2-4
Gas laws	3.24 ± 0.75	2-4
Friction	3.22 ± 0.81	2-4
Phototherapy	3.20 ± 0.78	2-4
Acceleration, speed and velocity,	3.20 ± 0.68	2-4
Gravity and centre of gravity	3.20 ± 0.68	2-4
Electricity – electrical conductivity	3.19 ± 0.83	2-4
Principles of MRI and issues	3.11 ± 0.76	2-4
Principles of Ultrasound and issues	3.11 ± 0.76	2-4
Principles of X-ray, CT, Fluoroscopy and issues	3.11 ± 0.81	2-4
Exothermic and endothermic reactions	3.08 ± 0.76	2-4
Matter - Atoms, isotopes, properties of elements, cations, anions, flammability of elements, effect of heat on matter	3.00 ± 0.79	2-4
Light - light sensors	3.00 ± 0.89	2-4
Force and motion, vectors	3.00 ± 0.76	2-4
Sound – auscultation and stethoscope use	3.00 ± .1.00	2-4

**Table 6:** **Topics ranked as low priority (below 3) by academics** ***(ranking: 1 = lowest priority to 5 = highest priority)***

**Topic**	**Mean/SD**	**Range**
**Chemistry**		
Chemical reactivity	2.95 ± .90	2-4
**Physics**		
Work/power mechanics - Traction, lever, energy and pulley	2.75 ± .86	2-4
Principles of Nuclear Medicine imaging, therapy and issues	2.94 ± .80	2-4
Principles of Radiotherapy and issues	2.94 ± .73	2-4

### Science topics rated as ‘high priority’

All items in Table 1 within the categories of basic concepts, anatomy and histology received ratings of up to 5. There was strong support for the teaching of anatomy, with 10 out of 11 items in this domain ranked high priority (mean ≥4.0). The eleventh item, gross anatomy of the reproductive system, was not given this level of priority in teaching.

When asked about teaching items relating to physiology and pathophysiology of body systems, there were diverse views overall. As can be seen in Table 2, only seven out of 86 items were seen as high priority in teaching as the remainder were ranked lower (<4). There was absolute agreement, however, that six of these seven items were high priority.

### Science topics rated as ‘moderate priority’

Respondents in the survey rated the majority of science topics as being given moderate priority in teaching (Tables 3, 4 and 5). In Table 3, eight out of ten elements of cellular histology featured as moderate priority, with only one of the anatomy items being included here.

Ratings for physiology and pathophysiology when clustered by body system received system-average ratings between 3.38 and 3.84 out of a possible five. Of these systems, those that featured most strongly in teaching were the renal system (overall mean = 3.84), respiratory system (M = 3.77) and endocrine system (M = 3.70). The lowest ratings overall were received by the reproductive system (M = 3.38) and genetics (M = 3.51).

All 22 microbiology topics, all but one chemistry topic (19 of 20) and the majority of physics topics (21 of 24) were ranked by respondents as moderate priority in teaching.

### Science topics rated as ‘low priority’

The respondents in this survey ranked very few science topics as low priority (i.e. below ‘3’) (Table 6). The only items that feature in this subset are from chemistry and physics. In the chemistry category, all but nine respondents thought teaching ‘chemical reactivity’ was a low priority. Around half the respondents rated the priority of a number of physics topics as low (ratings 1-2). Examples include ‘work/power mechanics - Traction, lever, energy and pulley’; ‘principles of nuclear medicine imaging, therapy and issues’; and ‘principles of radiotherapy and issues’

### Science topics not listed

Respondents were asked to indicate via a free-text option any items that were absent from the survey. Of those who took the opportunity to make comments in this section, most responded that the survey was comprehensive, with only topics of nutrition, breastfeeding and pharmacology listed.

### Difference between nurse and non-nurse academics

Exploratory analysis was conducted to determine whether nurse academics may hold different views about the priority given to science topics than science lecturers from other disciplines. Mann-Whitney *U* tests of the 177 science items confirmed there was no overall pattern of significant difference in prioritization between groups. Several individual items showed a trend of higher priority by the respondents - e.g., teaching the cardiovascular system and the respiratory system was seen as higher priority by non-nurse teachers, although this difference did not reach a level of significance (*p* = .06), and similarly for teaching about the neurological system (*p* = .09). Again the small sample size constrained analysis.

## Discussion

This study has revealed interesting information about the teaching of science to nursing students. In 2008, Logan identified the proportion of science taught in nursing programs to be around 16% [25]. Subsequent work by Logan and Angel [6] suggests that this figure is decreasing, although these authors acknowledge that science content is less distinctive and the true proportion of science may be masked in curricula that are becoming increasingly integrated. The number of academics teaching dedicated science content across the institutions that are accredited to conduct these programs in Australia is therefore similarly small, as reflected in the sample size in this study.

The results confirm that all topics contained within the survey are addressed in nursing programs. Earlier work has consistently indicated that the bioscience content of nursing programs is inadequate [1,24,27,28] yet few additional items were suggested by respondents when given the opportunity to do so. The identification of so few outstanding topic areas infers that academics either believe the science content of nursing programs *is* adequate or are unsure of what science content warrants inclusion in what is recognized to be an increasingly crowded curriculum [29].

Respondents ranked the majority of topics presented in the survey in the ‘moderate’ priority range. Most of the items that were ranked ‘high priority’ for teaching were macroscopic topics, such as gross anatomy and basic concepts above the cellular level. This finding is consistent with those of Davis who also found that anatomy was the bioscience topic given the greatest coverage in pre-registration curricula [24]. Topics such as microbiology and chemistry were noticeably not ranked in the ‘high priority’ category, once again reflecting the findings of Davis [24]. It is possible that concepts associated with more concrete elements of science such as anatomy, terminology etc., may be easier for students to grasp and thus easier to teach. It might also be the case that such subject areas are easier to deliver in a mass lecture or via online delivery thus reducing the resource requirements associated with the teaching of topics such as microbiology and physics.

The physiology and pathophysiology of all body systems was ranked ‘3’ or above. Of concern, however, is that with the exception of cardiovascular system, respiratory system and renal system, all these systems were ranked only as ‘moderate priority’. Effective and safe nursing practice is dependent upon highly developed assessment skills that can distinguish normal and deviations from normal. Furthermore, a comprehensive understanding of both physiology and pathophysiology is fundamental to the ability to accurately undertake nursing health assessment. Why the cardiovascular, respiratory and renal systems were the only systems identified as being given the highest priority in teaching is not clear. It is possible that in the case of a patient’s health deteriorating in an acute situation, an understanding of these systems is seen as most critical to informing a nursing response. The literature suggests that nurses’ collection and interpretation of clinical cues explained by these science domains is vital [29,30].

The reproductive system and genetics both ranked lowest in respect of the teaching of physiology and pathophysiology. It may be that respondents were focused on generic knowledge that was essential education for novice nurses, whereas reproduction could be considered the domain of more specialist disciplines, such as midwifery. With the exception of genetic control and malignancy, which was rated in the ‘high priority’ category, genetics was the lowest ranking topic in the category of physiology and pathophysiology. In spite of the increasing significance of genetics in ensuring a preparedness for practice from a contemporary evidence base [31], a generalist philosophy in undergraduate nursing curricula combined with a low number of specialist nurse geneticists in Australia may contribute to the low ranking of this category.

Few other topics in the survey ranked as ‘low priority’. Chemical reactivity may have been considered by respondents to be an overarching term for many of the items in the chemistry category. In spite of nursing practices no longer including lifting and moving patients, the low ranking of topics related to biomechanics of movement is concerning given the need for knowledge in this topic area remains a workplace health and safety issue. The low ranking of topics related to nuclear medicine and radiation may once again be considered areas of specialised practice and thus not seen as important for inclusion in a preparatory nursing program.

In approaching this study, the authors were acutely aware of a broad consensus in the literature that the bioscience content in nursing programs is inadequate. Ironically it would seem that the inclusion of science content in nursing curricula is being guided by a comprehensive philosophy in which quantity is emphasised over quality. Although a small sample size may limit the ability to draw conclusive inferences from the data, it is nevertheless surprising that so few topic areas were strongly identified as being given ‘high priority’ by respondents to this survey. In a study on bioscience content in nursing, Friedel and Treagust [1] identified a discrepancy between the intended and enacted curriculum, concluding that the aims of preparing students for clinical practice were not being fulfilled, a finding further validated by Davis [24]. The findings of these authors, along with those described in this paper, may indicate a problem with curriculum informants and decision-making processes that ultimately impact on curriculum content. The issues surrounding science content in nursing programs are therefore complex and require exploration of the large context in which curriculum development and delivery occur.

### Recommendations

A number of questions are raised by the preceding discussion that has implications for the profession: is the emphasis on a comprehensive philosophy of preparatory nursing education in Australia impeding the development of a solid foundation in the biological sciences? To what extent are these challenges the result of crowded curricula where the opportunity to pose solutions is being compounded by reducing academic teaching periods? Is the inadequacy in the teaching of science content in nursing reflective of a lack of decision-making responsibility in respect of curriculum design by key stakeholders?

Addressing these questions requires a commitment to understanding the drivers of educational practice in nursing and a rethinking of current practices in respect of nursing curricula. For example, while it may seem counterintuitive to improving the delivery of bioscience in nursing programs, a philosophy of “less is more” may rationalise the teaching of bioscience content and ensure that the content of curricula is clearly focused on preparing students for the demands of contemporary nursing practice. Similarly, the same philosophy may be applied to non-bioscience content as an increasing assortment of specialist nursing knowledge encroaches on curricula, leaving little room for the inclusion of science. Results of the subsequent phase of this research will inform such focus and further research is recommended to identify and adequately scope the issues related to academics’ teaching of bioscience in nursing programs. Academics responsible for the design and implementation of nursing curricula are encouraged to review the content of current programs in light of the findings of this and future work.

### Limitations

The major limitation of this study is the low sample size derived from a small population of science-teaching academics in Australia. This limitation has prohibited a more extensive analysis of the quantitative data. The sample is considered representative of the target cohort, with respondents located in all but one jurisdiction. Distribution of the survey link was at the discretion of the CDNM and subsequently the Deans and heads of schools of nursing nationwide. A potential limitation therefore relates to the constitution and responsiveness of the membership of the CDNM. A number of concepts may also have been subject to interpretation, for example: what constitutes ‘undergraduate nursing programs’ as referred to in the survey; what is considered to be science content; and who in any department is classified as a science teacher. In addition, there may be differences between the views of nursing academics and non-nursing academics, owing to their various educational backgrounds, that cannot be explicated as a result of the small sample size. The results nonetheless indicate that the responding cohort possess a comprehensive understanding of the science content of nursing curricula which can guide future curriculum review.

## Conclusion

It is interesting to note that a staple feature of nursing curricula such as science is hindered by an extant lack of research on what must be taught to provide the best preparation for students progressing towards nursing practice. The delivery of quality science content requires empirical evidence to ensure educational efficiency and contemporary relevance. The findings of this research support similar previous studies and go further in identifying specific issues that warrant consideration. Current approaches to delivering science in nursing programs should be reviewed in light of this evidence in order to prepare graduates to practice in a complex environment that requires flexible application of a diverse repertoire of knowledge and skills.
